# Overcoming the Constraints of Anti-HIV/CD89 Bispecific Antibodies That Limit Viral Inhibition

**DOI:** 10.1155/2016/9425172

**Published:** 2016-06-22

**Authors:** Xiaocong Yu, Mark Duval, Melissa Gawron, Marshall R. Posner, Lisa A. Cavacini

**Affiliations:** ^1^Department of Medicine, Beth Israel Deaconess Medical Center, Boston, MA 02215, USA; ^2^Harvard Medical School, Boston, MA 02215, USA

## Abstract

Innovative strategies are necessary to maximize the clinical application of HIV neutralizing antibodies. To this end, bispecific constructs of human antibody F240, reactive with well-conserved gp41 epitope and antibody 14A8, reactive with the IgA receptor (CD89) on effector cells, were constructed. A F240 × 14A8 bispecific single chain variable region (scFv) molecule was constructed by linking two scFvs using a conventional GGGGS linker. Despite immunoreactivity with HIV gp41 and neutrophils, this bispecific scFv failed to inhibit HIV infection. This is in sharp contrast to viral inhibition using a chemical conjugate of the Fab of these two antibodies. Therefore, we constructed two novel Fab-like bispecific antibody molecules centered on fusion of the IgG1 CH1 domain or CH1-hinge domain to the C-terminus of F240scFv and fusion of the kappa chain CL domain to the C-terminus of 14A8scFv. Both Bi-Fab antibodies showed significant ADCVI activity for multiple clade B and clade C isolates by arming the neutrophils to inhibit HIV infection. The approach presented in this study is unique for HIV immunotherapy in that the impetus of neutralization is to arm and mobilize PMN to destroy HIV and HIV infected cells.

## 1. Introduction

Notwithstanding the isolation of broadly neutralizing anti-HIV-1 human antibodies [1], there remain a number of limitations for clinical applications, including global subtype coverage. One approach is to broadly target virus using conserved nonneutralizing domains on the HIV-1 Env and to target virus for destruction using noninfectable effector cells. It has been shown that Antibody-Dependent Cellular Cytotoxicity (ADCC) can be mediated by nonneutralizing antibodies and it has been shown to be higher in HIV controllers [2, 3].

We previously demonstrated that a bispecific antibody, constructed by chemical conjugation of the Fab regions of F240 and the anti-CD89 (IgA receptor) antibody 14A8, promotes destruction of HIV by neutrophils [4]. F240 recognizes a highly conserved extracellular epitope (residues 598 to 604) on gp41 within cluster I and reacts with primary isolates from all clades of HIV-1 [5], similar to other cluster I antibodies. The majority of clades A, B, and C isolates in the HIV-1 sequence database have an identical peptide (amino acids 592 to 606), with the exception of clade D isolates, which have a consistent L602H mutation. Our prior study supports the notion that broadly reactive, nonneutralizing antibodies, such as F240, could be used to “neutralize” HIV and might be a practicable novel therapeutic strategy for prevention and treatment of HIV infection. However, chemical conjugation for the construction of bispecific antibodies is associated with technical and large scale production issues.

To create and study different bispecific molecular constructs and promote a better production process, we have constructed bispecific antibodies using conventional linkers of scFv fragments as well as two novel Fab-like bispecific antibody structures. Further, we demonstrate that specific recombinant bispecific antibody structures effectively inhibit HIV infection. The results described here also report on the contribution of molecular structure of the bispecific antibody to maximal anti-HIV functional activity.

## 2. Materials and Methods

### 2.1. Monoclonal Antibodies, Virus, and Cell Lines

Antibody F240, generated in our laboratory, binds to a broadly conserved domain of gp41 [5]. The 14A8 is a human anti-CD89 antibody that was generated in Medarex-Mouse human Ig transgenic mice [6]. The bispecific single chain (scFv) antibody expression vector pcDNA3.1 was from Invitrogen. The vectors of pLC-HuC*κ* and pHC-HuC*γ*1 for expressing Bi-Fab antibodies were obtained from Dr. McLean [7] and were modified for expressing Fab-like bispecific antibodies. CHO-K1 cells were from American Type Culture Collection. The following reagents were obtained through the AIDS Research and Reference Reagent Program, Division of AIDS, NIAID, NIH: SF162 (R5) from Dr. Jay Levy; BaL (R5) from Dr. Suzanne Gartner, Dr. Mikulas Popovic, and Dr. Robert Gallo; 93MW960 (clade C, R5) from Dr. Robert Bollinger and the UNAIDS Network for HIV; JR-FL (R5) from Dr. Irvin Chen; TZM-bl cells from Dr. John C. Kappes, Dr. Xiaoyun Wu, and Transzyme, Inc.; isolate 67970 (CXCR4) from Dr. David Montefiori.

### 2.2. Construction of Antibody F240 and 14A8 scFv Molecules

Total RNA was isolated from F240 hybridoma cell line and then amplified the variable domain fragments of heavy chain (VH) and light chain (VL) using RT-PCR. The 14A8 variable domain fragments of VH and VL were directly amplified using 14A8 antibody gene plasmids as PCR template. A peptide (GGGGS)_3_ was used as a linker to assemble VH and VL together to form the F240scFv and 14A8scFv individually by overlap PCR.

### 2.3. Construction of Conventional F240 × 14A8 Bis-scFv Antibody Plasmid and Establishing Stable Expressing Cell Lines

An F240 × 14A8 bispecific scFv antibody was constructed using a conventional structure. A DNA sequence that codes a short linker peptide consisting of glycine and serine residues (GGGGS) was designed to connect F240scFv and 14A8scFv by overlap PCR. The amplified Bi-scFv fragment was cloned into pcDNA3.1 vector utilizing the restriction sites of* NheI* and* HindIII*. The constructed plasmid was transfected into CHO cells with lipofectamine LTX (Invitrogen) using selection medium containing 1 mg/mL zeocin for at least two weeks. Limiting dilution at 1 cell per well was performed twice to obtain a stable cell line.

### 2.4. Constructing Plasmids of F240 × 14A8 Bi-Fab and Establishing Stable Expression Cell Lines

A human IgG CH1 fragment or CH1+ hinge fragment was amplified by PCR from the human IgG1 heavy chain and c-myc plus 6XHis tags was assembled into the 3′ end. The F240scFv genes (as described above with linkers) were connected directly with these amplified fragments individually by overlap extensional PCR, and the PCR products were cloned into the IgG expressing vector (Dr. McLean) to replace the human IgG1 constant domain. Meanwhile, the14A8scFv was cloned into the vector of pLC-HuC*κ* using* NheI/NotI* sites which contained human kappa chain constant. Paired purified plasmids encoding the 14A8scFv-CL versus F240scFv-CH1 or 14A8scFv-CL versus F240scFv-CH1-hinge were cotransfected into CHO-K1 cells in 6-well plates with lipofectamine LTX (Invitrogen Life Technologies). G418 (800 *µ*g/mL) and puromycin (10 *µ*g/mL) were used for selection of stable transfectants. The plates were screened using capture ELISA assay. The positive wells with top producing capacity were cloned by one-cell-per-well limiting dilution to establish the highest expressing cell clones. The expressed Bi-Fabs in culture supernatant were purified using protein L. Expression from the stable cell lines varied from a low expression of 1 *µ*g/mL to 50 *µ*g/mL depending on the tissue culture vessel used. Recovery of antibody following purification was similar to what we see using protein G columns and IgG and efficiency was proportional to the amount of antibody applied to the column up to capacity.

### 2.5. Immunoreactivity of F240 and CD89 Components of Bi-Fab Antibodies

To detect F240 reactivity, microplates were coated with recombinant gp41 (ectodomain aa 546–682 of HxB2 strain, Meridian) at 2 *µ*g/mL in PBS overnight at 4°C followed by blocking with BSA blocking buffer plus 0.01%-tween at room temperature for 2 hours. Bi-Fab samples were added at 1 *µ*g/mL and serial dilutions were performed. Samples were incubated for 30 minutes followed by washing and incubation with HRP-conjugated protein L (Pierce). The human monoclonal F240 was run as a standard to determine relative reactivity of the F240-14A8 Bi-Fabs. After washing, 100 *µ*L of TMB substrate was added and incubated for 5 minutes. Reaction was stopped with 100 *µ*L of 1 M phosphoric acid and plate was read on a plate reader at 450 nm.

Immunoreactivity of 14A8 with CD89 was determined by flow cytometry. Neutrophils were isolated from peripheral blood of HIV seronegative donors using Ficoll-Hypaque gradient centrifugation and dextran sedimentation. PMNs were washed twice with PBS and resuspended at a concentration of 1 × 10^7^ c/mL in HBSS containing 2.5% FBS. 100 *µ*L of PMNs was mixed with 100 *µ*L of serial diluted Bi-Fab antibodies and incubated on ice for 30 minutes. Cells were washed twice with PBS followed by 30-minute incubation with FITC-labeled Goat anti-human Ig Kappa (Southern Biotechnology Associates). PMNs were washed and fixed in 1% paraformaldehyde and samples were acquired and analyzed using Guava 8HT and Incyte software. Live cells were gated based on forward and side scatter.

### 2.6. Antibody-Dependent Cell-Mediated Viral Inhibition (ADCVI)

The ability of the antibody to arm neutrophils to inhibit HIV infection was measured as ADCVI activity of constructed bispecific antibodies using HIV grown in PHA stimulated PBMC [8, 9]. Bispecific antibodies were tittered in 96-well plates with 50 *µ*L media. Neutrophils (5 × 10^6^ cells/well) were added and incubated with antibody for 10 minutes. PBMC productively infected with HIV-1 four days earlier were used as target cells and 5 × 10^5^ infected cells were added to the antibody/effector cell mixture resulting in an E : T ratio of 10 : 1. The 10 : 1 E : T ratio was selected as the concentration of neutrophils that does not result in nonspecific inhibition of HIV by the neutrophils. After incubation for 4 hours, PHA stimulated PBMC (2 × 10^6^/well) were added as indicator cells for measuring the surviving infectious virus and incubated for seven days in the presence of IL-2 and p24 quantitated by ELISA [10]. Linear regression analysis was used to determine IC_50_ values and Student's *t*-test was used to detect significance. Control wells included absent antibody, absent effector cells, and absent target cells to determine background release of virus, maximal production of virus, and whether PMN alone were infected, respectively. Experiments were repeated three times.

## 3. Results

A bispecific single chain antibody was made using a G4S peptide to link the F240 scFv with the 14A8 scFv. The bispecific scFv was found to bind to both gp41 (ELISA) and CD89 on neutrophils (flow cytometry). However, in contrast to our results using a chemical conjugate of Fab fragments of F240 and 14A8^4^, the F240 × 14A8 biscFv antibody demonstrated limited ADCVI activity. At 10 *µ*g/mL, F240 × 14A8 biscFv only inhibited 21–23% for JR-FL or 89.6 and failed to inhibit 93MW960 (data not shown). At this concentration, the chemical conjugate of F240 and 14A8 inhibited more than 50% for the isolates tested above. To ensure that this was not due to a donor specific neutrophil defect, the experiment was repeated on four separate occasions using different donor neutrophils. While the biscFv antibody failed to mediate ADCVI, a positive control anti-HIV b12 IgA or A1g8-IgA antibody known to mediate ADCVI inhibited infection (>40% at 10 *µ*g/mL), in the presence of neutrophil effectors as previously reported [10]. Furthermore, failure of the bispecific scFv to mediate ADCVI was not related to immunoreactivity of the anti-gp41 F240 component as the sequence of the epitope on gp41 recognized by this antibody is identical in all isolates used in this study.

**Figure 1:**
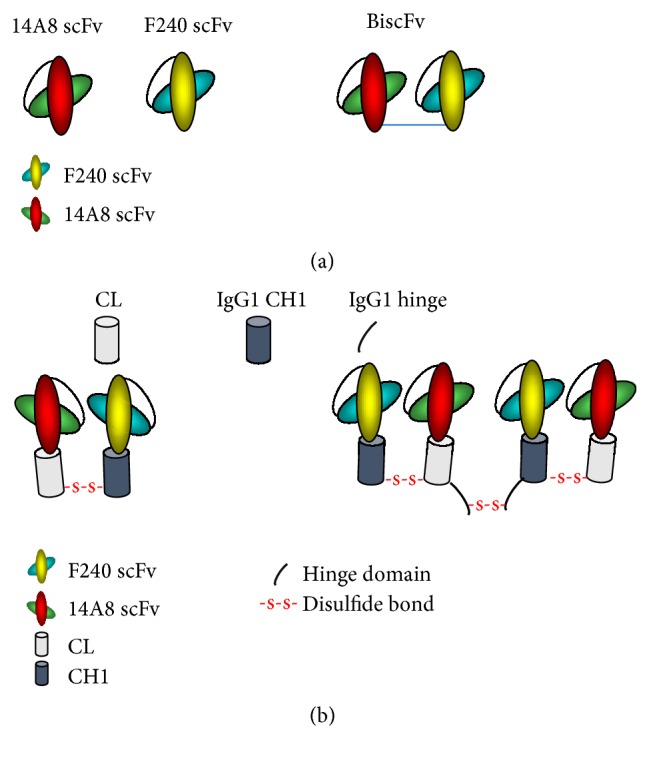
Schematic diagram of F240 × 14A8 Bi-Fab construction. The structure of the bispecific single chain antibody (biscFv) is depicted in (a) with the VH as either the green oval (14A8) or blue oval (F240) and the VL as the red oval (14A8) or yellow oval (F240). (b) represents the organization of the Bi-Fab.

**Figure 2:**
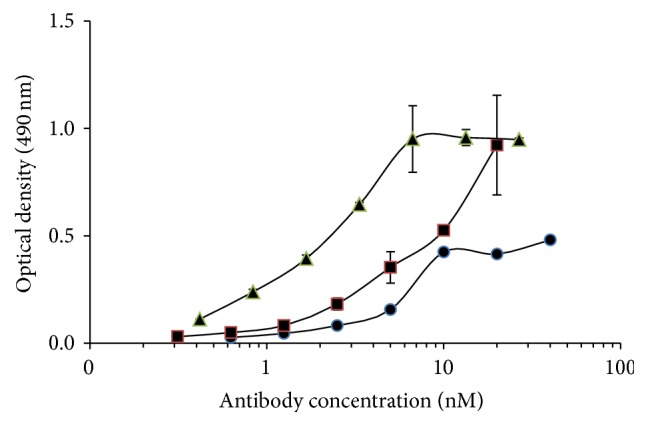
Immunoreaction of F240 × 14A8 Bi-Fab with gp41. Immunoreactivity of Bi-Fab antibody constructs with HIV antigen gp41. Antigen gp41 was coated at 2 *µ*g/mL in PBS; the serial dilutions of Bi-Fab antibodies (Bi-Fab A, ●, and Bi-Fab B, ■) compared to that of serial dilutions of F240 antibody (▲); the reaction was developed by HRP-conjugated protein L (1 : 20,000) and measured by optical density at 490 nm. Results are representative of three different experiments and each experimental point is the mean ± standard deviation of triplicate wells.

**Figure 3:**
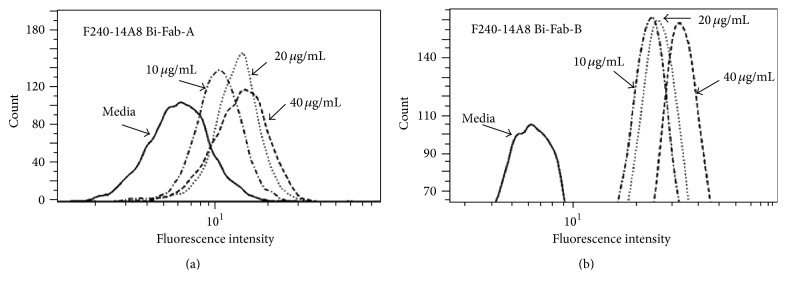
Binding affinity of F240 × 14A8 Bi-Fab with neutrophil through CD89. Neutrophils were washed twice with PBS and resuspended at a concentration of 1 × 10^7^ c/mL in HBSS containing 2.5% FBS. 100 *µ*L of neutrophils was mixed with 100 *µ*L of serial diluted Bi-Fab antibodies with Bi-Fab A in (a) and Bi-Fab B in (b). Cells were incubated with FITC-labeled goat anti-human Ig Kappa for 30 minutes. Neutrophils were fixed in 1% paraformaldehyde. Results are representative of four different neutrophil donors.

In reviewing the structure of the chemically conjugated versus biscFv bispecific antibodies, we hypothesized that failure of the biscFv to mediate ADCVI yet retain immunoreactivity may be a function of the structural constraints of the smaller and less flexible biscFv as compared to the Fab. The longer Fab fragment with the first Fc constant domain (CH1) and light chain constant (CL) may allow for more flexibility in bridging between the neutrophils and the infected cells. Therefore, we performed an innovative design to construct Fab-like bispecific antibodies. A dimeric or a tetrameric bispecific antibody was formed through the interchain disulfide bond between CH1 and CL or the CH1-hinge part and CL, respectively. The dimeric F240-CH1/14A8-CL is designated as F240 × 14A8 Bi-Fab-A and the tetrameric F240-CH1-hinge/14A8-CL as F240 × 14A8 Bi-Fab-B and as depicted in Figure 1. Immunoreactivity of the F240 × 14A8 Bi-Fab antibodies with HIV gp41 was determined by ELISA using F240 IgG1 antibody as the standard (Figure 2). Given that the epitope recognized by F240 is extremely conserved, it is expected that the Bi-Fab would react with the majority of isolates, with the exception of clade D. Both F240 × 14A8Bi-Fab antibodies react with gp41 and due to bivalency for gp41 binding, higher level binding is seen with Bi-Fab-B and the F240 IgG1, as compared to Bi-Fab-A. Immunoreactivity of the 14A8 component of Bi-Fab antibodies with CD89 is evident in Figure 3 using neutrophils from uninfected donors. Binding of both Bi-Fab antibodies occurs in a dose dependent manner and, similar to immunoreactivity with gp41, means that fluorescent intensity of Bi-Fab-B was greater than that seen for Bi-Fab-A.

Importantly, both Bi-Fab antibodies showed significant ADCVI activity for three clade B isolates and a clade C isolate (Table 1). There are significant differences in ADCVI activity among the virus isolates and between the different Bi-Fab constructs which is not necessarily explained by differences in the immunoreactivity with gp41 and neutrophils by the Bi-Fab molecules. Whereas both molecules were effective at inhibiting BaL (similar to the chemical conjugate of antibody Fabs), F240 × 14A8 Bi-Fab-A was more effective at ADCVI than F240 × 14A8 Bi-Fab-B for JR-FL. In contrast, the F240 × 14A8 Bi-Fab-B showed much higher ADCVI activity than Bi-Fab-A against 93MW960 and SF162 with the activity of the chemical conjugate intermediate between both of them. These differences may represent a number of mechanistic effects of the structure of both the antibody and the virus. Neither Bi-Fab construct neutralized HIV directly (data not shown).

## 4. Discussion

Building on our previous report of a chemically constructed bispecific antibody directing destruction of HIV^4^, we have produced molecular HIV specific bispecific antibody molecules incorporating the HIV gp41 specific antibody, F240, and an IgA receptor specific antibody, 14A8. A conventional design was used to link two scFvs for expression as a bispecific scFv. Despite immunoreactivity with HIV (gp41) and neutrophils, this bispecific scFv failed to mediate ADCVI activity. In retrospect, failure of the conventional F240 × 14A8 biscFv may be predicted by the structure of the biscFv. We hypothesized that failure of the biscFv to mediate ADCVI yet retain immunoreactivity with both HIV and neutrophils may be a function of the structural constraints of the scFv as compared to the Fab. The longer antibody Fab fragment with the first constant domain of Fc part may allow for more flexibility in bridging the neutrophils and the infected cells.

Viral isolates vary in quaternary structure such that env trimers may range from open to closed. Additional factors include differences in the spatial arrangement of envelope or the glycosylation pattern of viral isolates or the contribution of other membrane components as these studies were performed with HIV infected PBMC. Further studies using mutated viral isolates and crystallography are being designed to explore this. It is also evident that the structure of the bispecific antibody directly impacts function. Consistent with what was observed previously, the heavy chain CH1 domain can affect antibody binding affinity and fine specificity [11]. A larger, flexible structure conferred by the CH1 and CL domains significantly contributes to the action of the bispecific antibodies in inhibiting HIV. Whereas the single chain construct failed to function although it did bind HIV and PMN, a flexible Bi-Fab molecule was able to confer function. Interestingly, the activity of the chemical conjugate of the Fab of antibodies F240 and 14A8 is similar to both Bi-Fabs for BaL but intermediate between Bi-Fab A and Bi-Fab B for two other isolates (93MW960 and SF162). This would suggest that the structure or conformation of the variable regions (e.g., natural Fab versus scFv) also impacts function. Finally, it is clearly shown that cross-linking CD89, which occurs outside of the IgA binding site, is active even if CD89 is occupied by IgA and is a viable option to activate PMN to “inhibit” HIV. There is considerable research demonstrating that cross-linking of Fc*α*R (CD89) using bispecific antibodies can induce tumor cytotoxicity [12–15], as well as target pathogens, such as* Streptococcus pneumonia* [16],* Porphyromonas gingivalis* [17], and* Bordetella pertussis* [18]. The approach presented in this study is unique for HIV immunotherapy in that the impetus for “neutralization” is to arm and mobilize neutrophils, which do not get infected, to globally destroy HIV and HIV infected cells.

## 5. Conclusions

These results demonstrate that recombinant Fab-like bispecific antibody constructs effectively inhibit HIV. Moreover, the molecular bispecific antibody construct profoundly affects the functional activity of the antibody. The approach presented in this study is unique for HIV immunotherapy in that the impetus of neutralization is to arm and mobilize PMN to destroy HIV and HIV infected cells.

## Figures and Tables

**Table 1:** ADCVI activity of HIV-1 by F240 Bi-Fab antibody constructs.

	IC_50_ (*μ*g/mL)^a^			
	BaL	JR-FL	93MW960	SF162
	Clade B, R5	Clade B, R5	Clade C R5	Clade B, R5
Bi-Fab-A	3.38 ± 2.39	0.04 ± 0.04	27.50 ± 7.78	15.69 ± 5.49
Bi-Fab-B	4.43 ± 0.16	35.85 ± 5.87	1.93 ± 2.65	0.22 ± 0.04
	*p* = 0.60^b^	*p* = 0.01	*p* = 0.05	*p* = 0.06
Chemical bispecific^c^	4.35 ± 1.2	NT^d^	10.4 ± 7.7	10.7 ± 2.9

^a^The ADCVI activity was determined by IC_50_ that represents concentration (*μ*g/mL) of Bi-Fab antibody constructs required for 50% inhibition of HIV. Results are from three separate experiments.

^b^The *p* value reflects the comparison of Bi-Fab-A versus Bi-Fab-B.

^c^Chemical conjugation of Fab fragments of F240 and 14A8 as described in [4].

^d^NT: not tested.
